# Absolute Isotopic Abundance Ratios and Atomic Weight of a Reference Sample of Nickel

**DOI:** 10.6028/jres.094.034

**Published:** 1989

**Authors:** J. W. Gramlich, L. A. Machlan, I. L. Barnes, P. J. Paulsen

**Affiliations:** National Institute of Standards and Technology, Gaithersburg, MD 20899

**Keywords:** absolute ratio, assay, atomic weight, dimethylglyoxime, isotopic abundance, mass spectrometry, nickel

## Abstract

Absolute values have been obtained for the isotopic abundance ratios of a reference sample of nickel (Standard Reference Material 986), using thermal ionization mass spectrometry. Samples of known isotopic composition, prepared from nearly isotopically pure separated nickel isotopes, were used to calibrate the mass spectrometers. The resulting absolute isotopic ratios are: ^58^Ni/^60^Ni=2.596061±0.000728, ^61^Ni/^60^Ni=0.043469±0.000015,^62^Ni/^60^Ni=0.138600±0.000045, and ^64^Ni/^60^Ni=0.035295±0.000024, which yield atom percents of ^58^Ni=68.076886 ±0.005919, ^60^Ni = 26.223146±0.005144,^61^Ni=1.139894±0.000433, ^62^Ni =3.634528±0.001142, and ^64^Ni =0.925546±0.000599. The atomic weight calculated from this isotopic composition is 58.693353 ±0.000147. The indicated uncertainties are overall limits of error based on two standard deviations of the mean and allowances for the effects of known sources of possible systematic error.

## 1. Introduction

The Inorganic Analytical Research Division of the National Institute of Standards and Technology, has been conducting a long-term program of absolute isotopic abundance ratio and atomic weight determinations using high precision isotope ratio mass spectrometry. Previous atomic weight determinations include silver [1,2], chlorine [3], copper [4], bromine [5], chromium [6], magnesium [7], lead [8], boron [9], rubidium [10], rhenium [11], silicon [12], potassium [13], thallium [14], strontium [15], and gallium [16].

To obtain absolute isotopic ratios from the observed or relative measurements made on a mass spectrometer, it is necessary to calibrate the instrument using samples of accurately known isotopic ratios of the element under study. These synthetic isotopic standards, assayed and gravimetrically prepared from chemically pure and nearly isotopically pure isotopes, provide a bias correction (calculated isotopic ratio/observed isotopic ratio) which, when applied to the observed isotopic ratios of the reference sample being calibrated, allows absolute ratios to be calculated for the sample. The atomic weight is then obtained by multiplying the fractional abundance of each isotope by its nuclidic mass [17] and summing the resultant products. A more detailed description of the method used for the determination of isotopic abundance ratios and atomic weights at NIST is given elsewhere [2].

In 1961, the IUPAC Commission on Atomic Weights recommended a value of 58.71 for the atomic weight of nickel. That value was based on the isotopic abundance measurements of White and Cameron [18]. The Commission noted in its report that all chemical determinations that had been reported and believed to be significant gave a mean value for the atomic weight of 58.69. The best chemical determinations appeared in a series of publications by Baxter and associates in the 1920s [19,20,21]. These measurements yielded an average atomic weight of 58.694, with credibility increased by proof of accuracy of parallel work on the atomic weight of cobalt [22] which is now known accurately because of its mononuclidic state.

In 1973 the Commission reexamined both the chemical and mass spectrometric measurements and so recommended a lower value of 58.70 for the atomic weight of nickel. Later that year, Barnes et al. [23] published a superior but not absolute mass spectrometric measurement which produced an atomic weight value of 58.688, in good agreement with the chemical determinations. After reexamination in 1979, the Commission recommended the present standard atomic weight value of 58.69±0.01 [24]. Nickel is currently listed by the IUPAC Commission on Atomic Weights and Isotopic Abundances as one of the elements with an atomic weight with a large uncertainty [25].

Since no significant variations in the isotopic composition of nickel in nature have been observed, either in this study or in previous work, a high accuracy measurement of the atomic weight of a reference sample of nickel (SRM 986) will allow IUPAC to recommend a value with a much smaller uncertainty.

## 2. Experimental Procedure

### 2.1 Mass Spectrometry

Isotope ratio measurements were made on two different thermal ionization mass spectrometers with separate operators. One instrument was an NIST designed mass spectrometer equipped with a 30-cm radius of curvature, 90° magnetic sector (designated inst. #1, operator #1). The second instrument was a Finnigan-MAT 261 mass spectrometer^1^ (designated inst. #2, operator #2). The NIST instrument employed a shielded Faraday cage collector with a double slit collimator. The remainder of the measurement circuitry consisted of a parametric electrometer [26], a precision voltmeter, and a computer. Timing, magnetic field switching, and data acquisition were controlled by the computer. The Finnagin-MAT 261 is a 23-cm radius, 90° magnetic sector instrument which uses a non-normal entry and exit ion path. This arrangement gives the dispersion of an instrument of 2.78 times (64 cm) the radius. It is equipped with seven deep Faraday cup collectors, six of which are externally adjustable. Each cup has an individual amplifier contained within an evacuated, thermally controlled chamber. The chamber temperature is maintained to ±0.02 °C.

Nickel was thermally ionized from a platinum filament fabricated from 0.001×0.030 in (0.0025×0.076 cm) high purity platinum ribbon. Prior to filament fabrication, the platinum ribbon was heated for several hours in dilute hydrochloric acid to reduce any iron impurities that might cause isobaric interferences. After fabrication, the filaments were degassed for 1 h by passing a current of 3 A through them in a vacuum and under a potential field of 45 V. Filaments cleaned in this manner generally exhibited no detectable emissions for the nickel isotopes using blank filament loadings. The maximum background observed was 1.2×10^−16^ A at mass 58, which appeared to be a natural nickel background. This background would be insignificant for measurement of the natural standard and the point calibration mixes. Filaments used for measurements of the nickel separated isotopes were examined in the mass spectrometer prior to sample loading. Only those filaments which showed no detectable background were used for analysis of the separated isotopes.

All sample loadings were conducted in a Class 100 clean air hood. Pipets made of Pyrex tubing were used to transfer the samples from their containers to the filaments. The tubing was cleaned by heating in 8 mol/L HNO_3_ for 24 h, rinsing with ultra-high purity water, heating in 6 mol/L HCl for 24 h, followed by several rinsings in ultra-high purity water.

Samples were loaded onto the filaments under the following conditions: approximately 5 μg of solution (5 *μ*L of 1 mg/mL Ni as NiNO_3_ in 1+49 HNO_3_)^2^ was added to the filament and dried for 5 min at 1 A. Five *μ*L of a solution containing 17 mg Aerosil 300 (Degussa, Frankfurt, FGR) powder/g of solution, 0.34 mg/g AlCl_3_/g solution, and 0.1 g of high purity H_3_PO_4_/g of solution were added to the filament and dried at a current, through the filament, of 1, 1.3, and 1.5 A, each for a time period of 5 min. The filament was then slowly heated to fume off the excess H_3_PO_4_ and then heated for a few seconds at approximately 700 °C. After a 5 min cooling period, the filament was loaded into the mass spectrometer.

The analysis procedure for inst. #1 was as follows: the initial filament current was set to produce a filament temperature of 1000 °C. At 4 min intervals the filament temperature was increased by 50 °C until at 20 min a final temperature of 1250 °C was achieved. After measurement of baselines on both sides of each mass of interest, data were collected between 30 and 70 min into the analysis. For the reference material, 5 min sets of ratio measurements were made in the following order: 58/60, 61/60, 62/60, 64/60, 64/60, 62/60, 61/60, 58/60.

The analysis procedure for inst. #2 was slightly different. The heating pattern was the same with a maximum temperature of 1250 °C (as measured with a two color optical pyrometer) used. The six mass unit range covered by the nickel isotopes is just beyond the range of this instrument so that it was necessary to jump the magnetic field once to collect all of the ratios. Thus, the 58/60, 61/60, and 62/60 ratios were collected simultaneously during a 32 s integration, a jump of one mass unit was made and the 64/60 ratio was collected. Five sets of twenty such ratios were collected for each sample. The amplifiers were calibrated at the beginning of each sample run; baselines were collected at the beginning of each block of twenty ratios.

### 2.2 Purification of Separated Nickel Isotopes

Electromagnetically separated ^58^Ni, ^60^Ni, and ^62^Ni isotopes were obtained from the Nuclear Division, Oak Ridge National Laboratory. The ^58^Ni was designated sample 121426, the ^60^Ni was designated sample 121501, and the ^62^Ni was designated sample 158201. The certificate which accompanied each sample showed isotopic enrichment to approximately 99.8% for the ^58^Ni material, 99.8% for the ^60^Ni material, and 99.0% for the ^62^Ni material. The certificates included a semi-quantitative spectrographic analysis which showed that the principal impurities were Cu and Zn at the 0.1% level and several other elements at the 0.05% level.

To reduce these impurities to a level low enough so that they would not cause a significant error in either the assay procedure or the mass spectrometric ratio measurements, the separated isotopes were purified by a combination of cation exchange chromatography, ammonium hydroxide precipitation, electrodeposition and anion exchange chromatography.

Each separated isotope was treated as follows: the nickel (approximately 2 g of ^58^Ni and ^60^Ni, and 1.8 g of ^62^Ni) was dissolved in 20 mL of HNO_3_ (1 + 1), evaporated to dryness and then 25 g of HClO_4_ were added. The solution was evaporated to HClO_4_ fumes, and after cooling, 5 g of 10 mol/L HCl was added. This step was used to help eliminate any Cr that was present. The sample solution was evaporated to dryness and the residue was dissolved in 50 g of H_2_O. This solution was passed through two cation exchange columns (a single column of the necessary length was not available) in series (each column, 19×1.6 cm, was filled with AG 50×8, 100–200 mesh resin and cleaned with 120 g of 4 mol/L HC1 followed with H_2_O until the eluate was neutral). After adding the nickel, some impurities were eluted from the columns with 200 g of 0.4 mol/L HCl. The nickel was eluted with 120 g of 3 mol/L HCl and this solution was evaporated to dryness. The residue was dissolved in 50 g of H_2_O, an excess of NH_4_OH was added (approximately 20 mL) and the solution was filtered to remove insoluble hydroxides. The solution was evaporated to dryness, 25 g of H_2_O were added and then 25 g of H_2_SO_4_ were slowly added. The solution was evaporated to fumes of H_2_SO_4_ and, after cooling, 5 g of HNO_3_ were added. The beaker was covered and heated, then the cover was removed and the solution evaporated to fumes of H_2_SO_4_. The sides of the beaker were rinsed down with H_2_O and the solution again evaporated to fumes of H_2_SO_4_. The solution was diluted to 150 mL with H_2_O, two clean platinum gauze electrodes were placed in the solution and a 2 V dc potential was applied between the electrodes for 16 h. The electrodes were removed from the solution and re-cleaned in HNO_3_ (1 + 1). The electrodes were placed back into the separated isotope solution, NH_4_OH was added to approximately 20 mL excess and a 2.0 V dc potential was applied until the nickel color had disappeared from the solution (this required up to three days, with an additional 5 mL of NH_4_OH being added each day). The electrode with the nickel deposit was removed from the solution, rinsed with H_2_O and the nickel was dissolved by heating in 40 g of 10 mol/L HNO_3_. The nickel solution was evaporated to dryness, 20 g of 5 mol/L HCl were added and the solution was again evaporated to dryness. The 5 mol/L HCl addition and evaporation to dryness was repeated. The residue was dissolved in 10 g of 5 mol/L HCl and all but 4 g of this was evaporated. Thirty g of 9.5 mol/L HCl were added and the resulting solution was passed through two anion exchange columns in series (each column consisted of a 5-mL plastic syringe filled to 5 mL with AG1×8, 100–200 mesh resin and cleaned with 40 g of 9 mol/L HCl, 50 g of H_2_O, and 10 g of 9 mol/L HCl). Ten g of 9 mol/L HCl were used to rinse the beaker and complete the elution of the nickel from the column. The sample was converted to the nitrate form by adding sequentially 20, 15, and 10 g portions of HNO_3_ to the beaker and evaporating the sample to dryness between each addition of acid.

### 2.3 Preparation and Analysis of Separated Isotope Solutions

The purified ^58^Ni and ^60^Ni were transferred to 500-mL Teflon bottles and diluted to approximately 400 g with HNO_3_ (1 +49). The purified ^62^Ni was transferred to a 2-L Teflon bottle and diluted to approximately 1700 g with HNO_3_ (1+49).

A preliminary assay of the nickel concentration of each separated isotope solution was accomplished by isotope dilution mass spectrometry. Two weighed portions of the ^60^Ni separated isotope solution were spiked with known amounts of natural nickel. Two weighed portions of each of the other two separated isotopes (^58^Ni and ^62^Ni) were spiked with weighed portions of the ^60^Ni separated isotope. After mixing, evaporation and dilution to 1 mg Ni/g of solution with HNO_3_ (1+49), the ^58^Ni/^60^Ni or ^62^Ni/^60^Ni ratios were determined by thermal ionization mass spectrometry. The concentration of nickel was then calculated for each solution and used to determine the amount of each separated isotope solution required for the calibration mixes. Samples of the three separated isotope solutions were analyzed for impurity elements by inductively coupled plasma mass spectrometry (ICP-MS).

Samples, equivalent to approximately 1.5 mg of Ni, were spiked with 1.5 ×l0^–8^ g each of ^206^Pb, ^203^Tl ^201^Hg, ^195^Pt; ^183^W, ^145^Nd, ^142^Ce, ^137^Ba, ^125^Te, ^123^Sb, ^113^In, ^111^Cd, ^110^Pd, ^97^Mo, ^91^Zr, ^87^Rb, ^86^Sr, ^73^Ge, ^71^Ga, ^67^Zn, ^65^Cu, ^57^Fe, “Cr, ^50^V, ^47^Ti, ^26^Mg, and ^6^Li. The solutions were diluted to 1 mg Ni/mL with HNO_3_ (1+49). Table 1 shows the results of these isotope dilution analyses as well as concentrations estimated from relative sensitivity factors (rsf). The rsf values are derived from a mass vs sensitivity response curve obtained from an external standard containing 20 elements spaced across the mass scale from Li to Hg. Table 2 shows the results of the ICP-MS analyses of SRM 986 and the same nickel material doped with 0.1 % of natural Pb, Au, Pt, Ba, Cd, Pb, Zn, Cu, Co, Fe, Mn, Cr, Ca, Al, and Mg, and then cleaned up with the same separation procedure developed for the separated nickel isotopes. The pure nickel was analyzed by a combination of stable isotope dilution and comparison to an external standard. The doped and cleaned sample was analyzed only by comparison to the external standard. The external standard was made by adding 10 ppm (10 μg/g Ni) of each of the doping elements to the pure nickel solution, thus producing a matrix matched standard.

### 2.4 Assay of Separated Isotope Solutions

Four weighed portions containing approximately 1.7 mmol of nickel were withdrawn from each separated isotope solution in the following manner: a polyethylene stopper with a 20-cm Teflon needle inserted through it was used to replace the cap on the bottle. A 20-mL all polypropylene—polyethylene syringe was attached to the hub of the needle and the desired amount of solution was withdrawn. The syringe was then disconnected from the hub and the tip was capped with a plastic cap. Any static charge that might be present on the plastic syringe was dissipated by wiping it with a damp lintless towel and placing it on the balance pan that was surrounded by several polonium anti-static sources. The syringe and contents were weighed on a semimicro balance to ±0.02 mg. The solution was then delivered from the syringe into a 600-mL pyrex beaker and the syringe was again capped, wiped and weighed. The weight of the sample was determined from the weight of the syringe before and after the delivery of the sample. Two assay samples were withdrawn from each solution before after the calibration samples and two after to ensure that no change in concentration occurred during the time interval (about 6 h) required for the aliquanting. Each weighed sample was assayed as follows: the sample was evaporated to dryness and converted to the chloride by adding 10 mL of 4 mol/L HCl and evaporating slowly to dryness. The addition of 10 mL of 4 mol/L HCl and the evaporation were repeated two more times. Two g of 4 mol/L HCl, 4 g of ammonium citrate solution (prepared by dissolving 25 g of (NH_4_)_2_HC_2_H_5_O_7_ in 200 mL of water, filtering and diluting with water to 250 mL) and 250 mL of water were added to the sample. A weighed portion of dimethylglyoxime reagent solution (prepared by dissolving 10 g of dimethylglyoxime in n-propanol, filtering and diluting with n-propanol to 1 L) equal to the amount required to form nickel dimethylglyoxime and 10 g excess was added to each assay sample. Ten g of dimethylglyoxime reagent solution and 30 g of n-propanol were added to each blank. The sample was heated in a water bath maintained at 65±2 °C. Five drops of 4 mol/L NH_4_OH were added, the sample was stirred with a glass stirring rod and after 5 min the addition of ammonia and stirring were repeated. The addition of 4M NH_4_OH was repeated, gradually increasing the number of drops to ten, until the Ni was completely precipitated as determined by the solution remaining colorless with the addition of NH_4_OH. Three more additions of ten drops of ammonia were made to ensure the precipitation of the nickel. The sample was removed from the water bath 30 min after the last ammonia addition. A total time of approximately 3 h was required for the precipitation. The nickel dimethylglyoxime crystals that are formed by this procedure are relatively large when compared to the usual method of precipitation.

The sample was allowed to stand approximately 48 h and then was filtered through a tared 15 mL glass filtering crucible of medium porosity. As much of the nickel dimethylglyoxime crystals as possible was transferred to the crucible using a water wash. The material in the crucible was washed three times with water (a total of 55–60 g of water was used for transfer and washing). The crucible and contents were dried at 150 °C for 16 h. The filtrate was transferred back to the original beaker and reserved for the determination of dissolved and untransferred nickel.

The filtering crucible and contents were cooled in a desiccator, transferred to the case of a mi-crobalance and allowed to stand for at least 2 h. The crucible and contents were weighed to ±0.002 mg. A combination blank and buoyancy correction was made by averaging three crucibles that had been used to filter blank samples which had been carried through the procedure. The crucible and contents were dried an additional 3 h at 150 °C and the cooling and weighing were repeated. The additional drying, cooling and weighing were repeated until constant weight was reached. The air weight of the nickel dimethylgly-oxime was then determined and converted to vacuum weight using 1.606 for the density of ^58^NiC_8_H_14_O_4_N_4_, 1.617 for the density of ^60^NiC_8_H_14_O_4_N_4_, and 1.628 for the density of ^62^NiC_8_H_14_O_4_N_4_. These densities were calculated by assuming that they were proportional to the density of natural NiC_8_H_14_O_4_N_4_, 1.611, in the same relationship as their molecular weight. The vacuum weight of the nickel dimethylglyoxime was converted to millimoles of nickel using a calculated atomic weight for nickel and the 1987 atomic weight values for carbon, hydrogen, oxygen, and nitrogen. The formula weights used were 288.1616 for the ^58^NiC_8_H_14_O_4_N_4_, 290.1511 for ^60^NiC_8_H_14_O_4_N_4_ and 292.1279 for ^62^NiC_8_H_14_O_4_N_4_.

The filtrate from the precipitation of the ^62^NiC_8_H_14_O_4_N_4_ was spiked with approximately 0.5 *µ*mol of ^60^Ni and the filtrate from the ^58^Ni and ^60^Ni precipitation were spiked for determining soluble and untransferred nickel by isotope dilution mass spectrometry with approximately 0.5 *µ*mol of ^62^Ni. After adding the spike, the pH of the filtrate solution was adjusted to 1.65±0.05 with 4 mol/L HCl. The solution was heated for 2 h to ensure equilibration of the spike and sample nickel. After cooling, this solution was passed through a cation exchange column (7.0×0.45 cm filled to 4.5 cm with AG 50×8, 100–200 mesh cation exchange resin and cleaned with 25 g of 4 mol/L HCl and H_2_O until the eluate was neutral), washed with a few mL of H_2_O and then 150 mL of 0,25 mol/L HCl. The nickel was eluted with 18 g of 4 mol/L HCl and the eluate was evaporated to dryness on a hot plate. A few drops of HNO_3_ and HClO_4_ were added to the residue and it was heated to help decompose the organic material. The sample was evaporated to dryness, cooled and the residue dissolved in 10 g of H_2_O. This solution was passed through the same cation exchange column after cleaning the column as before and washed with a few mL of H_2_O and 20 g of 0.25 mol/L HCl. The nickel was eluted with 18 g of 4 mol/L HCl and the eluate was evaporated to dryness on a hot plate. A few drops of HNO_3_ and HClO_4_ were added to the residue and it was heated to dryness. The residue was dissolved in a few drops of HNO_3_ (1+49) and the nickel isotopic ratios were determined by thermal ionization mass spectrometry. The nickel found as soluble Ni was added to the nickel determined by gravimetry to yield the total nickel in the sample. Table 3 shows the results of these analyses.

This method of determining the concentration of nickel solutions was previously tested on solutions containing known amounts of nickel. Solutions were prepared from high purity nickel metal. The nickel concentration in five sets of four samples, each containing 1.58 to 1.79 mmol of nickel was determined as described above. Comparison of the calculated and measured concentrations detected a positive bias of about 0.03 percent, but this would have a negligible effect on ratios.

### 2.5 Isotopic Analysis of the Separated Isotope Solutions

Each of the three separated isotope solutions was analyzed eight times by operator # 1 on instrument #1. The ion source was cleaned between analyses of the solutions as a precaution against the possibility of cross-contamination from the source parts. Preliminary measurements showed that the different separated isotope solutions could be analyzed back-to-back on the same source with no detectable cross-contamination.

As mentioned in section 2.1, preliminary measurements to evaluate possible sources of systematic errors indicated that a small natural nickel background could be present on some filaments. Acid leaching of the filaments and optimized filament outgassing procedures minimized the magnitude and frequency of this problem. This optimization was achieved by monitoring the ^58^Ni on increasingly smaller sample sizes of the ^60^Ni separated isotope, and measuring the nickel signal from filaments cleaned and degassed under various conditions using an ion counting detection system. All filaments used for measurement of the separated isotopes were examined in the mass spectrometer prior to sample loading. In addition to monitoring all nickel masses for contamination, ^56^Fe and ^66^Zn were examined to insure the absence of isobaric interferences. Only those filaments which showed no detectable (<l×10^−16^A) signal were used for the measurement of the separated isotopes.

The corrected isotopic compositions of the separated isotopes are given in Table 4.

### 2.6 Preparation of Calibration Samples

Five calibration samples were prepared by mixing weighed portions of “Ni-58”, “Ni-60”, and “Ni-62” solutions. The portions were withdrawn from the bottles and weighed in the manner previously described for the assay of the solutions. The portions weighed from 4.9 to 110 g and each was weighed to ±0.05 mg. It is therefore estimated that the weighing error for each mix should not exceed two parts in 10^5^. To minimize any significant possibility of change in concentration of the isotope solutions with time, the portions for the calibration mixes were withdrawn from the bottles between the samples taken for assay, over a period of about 6 h.

Each calibration mix was thoroughly mixed, the sides of the beaker were washed with H_2_O and 0.3 mol/L HNO_3_ and evaporated to dryness on a hot plate. The residue was dissolved and diluted with HNO_3_ (1+49) to 5 mg Ni per gram of solution. After thorough mixing, a portion of this solution was diluted with HNO_3_ (1+49) to 1 mg Ni per gram of solution and transferred to a small Teflon bottle. The isotopic compositions of the calibration mixes are given in table 5.

### 2.7 Isotopic Analyses of the Calibration Mixes and the Reference Sample

Two sets of analyses of the calibration mixes and reference sample were performed on instrument # 1 and instrument #2. Instrument #1 was used to measure 28 samples of the reference material and 4 samples each of mixes 1–5. Instrument #2 was used to measure 21 samples of the reference material and 6 samples of mixes 1–5. The samples were analyzed in a random pattern of mixes and the reference sample. Deviations from a totally random pattern were the initial successive analyses of the reference material to insure instrument and statistical control and the requirement that the same mix solution not be analyzed in succession.

## 3. Results and Discussion

The results of the measurements of the calibration mixes are shown in Table 6. Table 7 summarizes the observed and corrected nickel isotopic ratios for the reference sample for operators 1 and 2 respectively, as well as the absolute isotopic abundance ratios for nickel and their uncertainties.

Table 8 gives summary calculations for the reference sample. The atomic weight is calculated from the absolute isotopic abundance by summing the product of the nuclidic masses [17] and the corresponding atom fractions.

As mentioned in the introduction, the presently recommended atomic weight of nickel is 58.69±0.01. The IUPAC Commission on Atomic Weights and Isotopic Abundances lists this as one of the least well known atomic weights and also one of the few remaining elements where the atomic weight is based, at least in part, on chemical determinations made in the early 1920s. Based on this work, a value of 58.6934±0.0002 will be recommended which is two orders of magnitude more precise and, more importantly, is now known on an absolute scale.

The reference material is issued by the NIST Office of Standard Reference Materials as SRM 986, Nickel Metal Isotopic Standard, and is certified for isotopic composition.

## Figures and Tables

**Table 1:** Analysis of impurities in the nickel separated isotopes

Element	Spike isotope	^58^Ni	^60^Ni	^62^Ni

Concentrations in μg/g, determined by isotope dilution				
Pb	^206^Pb	1.3	2.2	3.1
T1	^203^T1	0.1	0.1	0.3
Hg	^201^Hg	6	2	2
Pt	^195^Pt	6.5	9.4	15
W	^183^W	0.9	2	1
Nd	^145^Nd	0.1	1	0.1
Ce	^142^Ce	0.1	0.2	0.3
Ba	^137^Ba	0.1	0.2	0.2
Te	^125^Te	0.6	0.5	2
Sb	^123^Sb	0.9	1	0.1
In	^113^In	0.1	0.5	0.1
Cd	^111^Cd	0.9	2	3
Pd	^110^Pd	1.6	2	2
Mo	^97^Mo	1.5	0.5	0.7
Zr	^91^Zr	2	2	0.5
Sr	^86^Sr	0.1	0.2	0.2
Rb	^87^Rb	0.3	0.4	0.2
Ga	^71^Ga	0.3	1	0.4
Ge	^73^Ge	2	2	1
Zn	^67^Zn	0.3	≤5	1
Cu	^65^Cu	2	≤12	a
Fe	^57^Fe	a	≤35	≤16
Cr	^53^Cr	0.7	0.4	0.6
V	^50^V	3	1	1
Ti	^47^Ti	3	3	3
Mg	^26^Mg	3	4	1
Li	^6^Li	≤14	≤15	≤20

Concentrations in μg/g, determined by rsf and/or external std				
U		0.1	0.1	0.1
Th		0.1	0.1	0.1
Bi		0.1	0.1	0.1
Au		0.2	0.2	0.2
Ir-I		0.3	0.3	0.3
Sn		0.2	0.9	0.8
Ag		0.3	0.1	0.1
Rh		0.3	0.1	0.1
Ru		0.3	0.3	0.3
Y		0.3	0.1	0.1
Se		1	4	1
As		1	1	1
Co^b^		≤240	≤2000	13
Mn		1	2	1
Sc		1	1	0.1
A1		≤3	≤3	≤3
Na		≤10	≤10	≤10
B		1	1	1
Be		1	1	1

a Matrix interference.

b ^58^Ni and ^60^Ni tails at ^59^Co.

**Table 2:** Analysis of impurities in the pure nickel (SRM 986) and the doped and purified nickel standard

Element	Pure nickel	Doped and purified Ni

	Isotope dilution	External standard in Ni
Pb	≤0.4	2
Tl	≤0.2	≤0.2
Nd	≤0.8	≤0.7
Ba	≤0.4	9
Te	≤0.6	≤0.5
Sn	2	0.6
Cd	≤0.3	≤0.2
Pd	9	≤0.1
Sr	≤0.1	≤0.1
Se	≤2	≤2
Zn	≤0.7	≤0.7
Cu	≤3	≤1
Cr	0.7	76
Mg	≤0.1	4

External standard in Ni		
Au	≤2	≤2
Pt	≤0.8	17
Co	≤4	≤4
Mn	0.4	9
Ca	≤4	≤4
A1	4	11

**Table 3:** Concentration of nickel in separated isotope solutions

Solution	Sample no.	Weight NiDMG^a^ (g)	Ni from NiDMG (mmol)	Ni from filtrate (mmol)	Total Ni (mmol)	Weight sample (g)	Concentration (mmol Ni/g)
“Ni-58”	1	0.487793	1.692775	0.000444	1.693219	19.64900	0.0861733
	2	0.492528	1.709207	0.000480	1.709687	19.83881	0.0861789
	3	0.486624	1.688720	0.000494	1.689214	19.60097	0.0861801
	4	0.488016	1.693550	0.000544	1.694094	19.65837	0.0861767
						Total	0.0861773
						SD	0.0000030
“Ni-60”	1	0.506874	1.746931	0.000449	1.747380	20.31729	0.0860046
	2	0.497094	1.713225	0.000559	1.713784	19.92565	0.0860089
	3	0.501435	1.728185	0.000451	1.728636	20.09839	0.0860087
	4	0.599604	1.725323	0.000563	1.725323	20.06610	0.0860100
						Total	0.0860081
						SD	0.0000024
“Ni-62”	1	0.509754	1.744969	0.000397	1.745366	101.53728	0.01711267
	2	0.512447	1.754186	0.000465	1.754651	102.53728	0.01711232
	3	0.510848	1.748713	0.000458	1.749171	102.21630	0.01711245
	4	0.511917	1.752374	0.000497	1.752871	102.43149	0.01711264
						Total	0.01711253
						SD	0.00000017

a NiDMG=nickel dimethylglyoxime.

**Table 4:** Isotopic composition of separated nickel isotopes used in calibration samples

Separated isotope	Isotopic composition		
	Atom percent		2-sigma uncertainty
“Ni-58”	58	99.873684	0.000478
	60	0.116721	0.000308
	61	0.002126	0.000156
	62	0.005364	0.000238
	64	0.002105	0.000164
“Ni-60”	58	0.226876	0.000700
	60	99.702709	0.001176
	61	0.027175	0.000528
	62	0.033806	0.000256
	64	0.009434	0.000456
“Ni-62”	58	0.355954	0.000454
	60	0.478222	0.000698
	61	0.096433	0.000438
	62	99.007380	0.001272
	64	0.062011	0.000152

**Table 5:** Isotopic composition of calibration samples

Solution no.	Isotope solution	Weight solution (g)	Ni from solution (mmol)	^58^Ni from solution (mmol)	^60^Ni from solution (mmol)	^62^Ni from solution (mmol)	Total^58^Ni (mmol)	Total ^60^Ni (mmol)	Total ^62^Ni (mmol)	Ratio ^58^Ni/^60^Ni	Ratio ^62^Ni/^60^Ni
1	“Ni-58”	19.16757	1.651809	1.649723	0.001928	0.000089					
	“Ni-60”	7.22959	0.621803	0.001411	0.619955	0.000210					
	“Ni-62”	5.00563	0.085659	0.000305	0.000410	0.084809					
							1.651439	0.622292	0.085107	2.653798	0.136764
2	“Ni-58”	18.41320	1.586800	1.584795	0.001852	0.000085					
	“Ni-60”	7.02949	0.604593	0.001372	0.602796	0.000204					
	“Ni-62”	5.07309	0.086813	0.000309	0.000415	0.085951					
							1.586476	0.605063	0.086241	2.622002	0.142532
3	“Ni-58”	18.46549	1.591306	1.589296	0.001857	0.000085					
	“Ni-60”	7.12359	0.612686	0.001390	0.610865	0.000207					
	“Ni-62”	5.00786	0.085697	0.000305	0.000410	0.084846					
							1.590991	0.613132	0.085139	2,594858	0.138859
4	“Ni-58”	18.42601	1.587904	1.585898	0.001853	0.000085					
	“Ni-60”	7.19758	0.619050	0.001404	0.617210	0.000209					
	“Ni-62”	4.93759	0.084494	0.000301	0.000404	0.083656					
							1.587603	0.619467	0.083950	2.562852	0.135520
5	“Ni-58”	18.11755	1.561322	1.559349	0.001822	0,000084					
	“Ni-60”	7.14866	0.614843	0.001395	0.613015	0.000208					
	“Ni-62”	5.08622	0.087038	0.000310	0.000416	0.086174					
							1.561054	0.615253	0.086466	2.537254	0.140536

**Table 6:** Determination of mass spectrometer bias

Calibration sample no.	Calculated		Isotopic ratios				Correction factors			
			Operator 1		Operator 2		Operator 1		Operator 2	
	^58^Ni/^60^Ni	^62^Ni/^60^Ni	^58^Ni/^60^Ni	^62^Ni/^60^Ni	^58^Ni/^60^Ni	^62^Ni/^60^Ni	^58^Ni/^60^Ni	^62^Ni/^60^Ni	^58^Ni/^60^Ni	^62^Ni/^60^Ni
1	2.653798	0.136764	2.695878	0.134686	2.694002	0.134730	0.984391	1.015430	0.985077	1.015102
2	2.622002	0.142532	2.663804	0.140356	2.659318	0.140512	0.984307	1.015506	0.985968	1.014378
3	2.594858	0.138859	2.635760	0.136696	2.633919	0.136854	0.984483	1.015826	0.985170	1.014651
4	2.562852	0.135520	2.604370	0.133421	2.600775	0.133575	0.984056	1.015734	0.985419	1.014563
5	2.537254	0.140536	2.577182	0.138349	2.575263	0.138502	0.984508	1.015817	0.985241	1.014687
Average correction factors							0.984349	1.015663	0.985375	1.014676
Correction factor standard deviations							0.000182	0.000183	0.000354	0.000267

**Table 7:** Corrected isotopic ratios of the SRM

Ratio	Operator 1	Operator 2	Average ratio
^58^Ni/^60^Ni	2.5961255	2.5959968	2.5960612
^61^Ni/^60^Ni	0.0434680	0.0434703	0.0434691
^62^Ni/^60^Ni	0.1385976	0.1386025	0.1386001
^64^Ni/^60^Ni	0.0353113	0.0352784	0.0352949

**Table 8:** Atomic weight, atom percent, and isotopic ratios of nickel

	Value	Total uncertainty (2-sigma)	Due to uncertainty in assay (2-sigma)	Due to uncertainty in mass spectrometry (2-sigma)	Due to uncertainty in mix preparation (2-sigma)	Due to uncertainty in nuclidic mass (2-sigma)
Atomic weight	58.693353	0.000147	0.000091	0.000104	0.000049	0.000002
Atom percent						
^58^Ni	68.076886	0.005919	0.004045	0.003762	0.002125	
^60^Ni	26.223146	0.005144	0.003776	0.002724	0.002186	
^61^Ni	1.139894	0.000433	0.000138	0.000402	0.000083	
^62^Ni	3.634528	0.001142	0.000615	0.000817	0.000508	
^64^Ni	0.925546	0.000599	0.000317	0.000420	0.000287	
Isotopic ratios						
^58^Ni/^60^Ni	2.596061	0.000728	0.000525	0.000410	0.000293	
^61^Ni/^60^Ni	0.043469	0.000015	0.000004	0.000014	0.000004	
^62^Ni/^60^Ni	0.138600	0.000045	0.000028	0.000025	0.000025	
^64^Ni/^60^Ni	0.035295	0.000024	0.000014	0.000015	0.000013	

## Footnotes

1 Certain commercial equipment, instruments, or materials are identified in this paper to specify adequately the experimental procedure. Such identification does not imply recommendation or endorsement by the National Institute of Standards and Technology, nor does it imply that the materials or equipment identified are necessarily the best available for the purpose.

2 A reagent dilution of (1 + 49) indicates 1 volume of concentrated reagent diluted with 49 volumes of pure water. If no dilution is specified, use of concentrated reagents is implied. The acids and water used for these dilutions were produced at NIST by sub-boiling distillation [26].

## References

[1] Shields WR, Garner EL, Dibeler VH (1962). J Res Natl Bur Stand (US).

[2] Powell LJ, Murphy TJ, Gramlich JW (1982). J Res Natl Bur Stand (US).

[3] Shields WR, Murphy TJ, Garner EL, Dibeler VH (1962). J Amer Chem Soc.

[4] Shields WR, Murphy TJ, Garner EL (1964). J Res Natl Bur Stand (US).

[5] Catanzaro EJ, Murphy TJ, Garner EL, Shields WR (1964). J Res Natl Bur Stand (US).

[6] Shields WR, Murphy TJ, Catanzaro EJ, Garner EL (1966). J Res Natl Bur Stand (US).

[7] Catanzaro EJ, Murphy TJ, Garner EL, Shields WR (1966). J Res Natl Bur Stand (US).

[8] Catanzaro EJ, Murphy TJ, Shields WR, Garner EL (1968). J Res Natl Bur Stand (US).

[9] Catanzaro EJ, Champion CE, Garner EL, Marinenko G, Sappenfield KM, Shields WR (1970).

[10] Catanzaro EJ, Murphy TL, Garner EL, Shields WR (1969). J Res Natl Bur Stand (US).

[11] Gramlich JW, Murphy TJ, Garner EL, Shields WR (1973). J Res Natl Bur Stand (US).

[12] Barnes IL, Moore LJ, Machlan LA, Murphy TJ, Shields WR (1975). J Res Natl Bur Stand (US).

[13] Garner EL, Murphy TJ, Gramlich JW, Paulsen PJ, Barnes IL (1975). J Res Natl Bur Stand (US).

[14] Dunstan LP, Gramlich JW, Barnes IL, Purdy WC (1980). J Res Natl Bur Stand (US).

[15] Moore LJ, Murphy TJ, Barnes IL (1982). J Res Natl Bur Stand (US).

[16] Machlan LA, Gramlich JW, Powell LJ, Lambert GM (1986). J Res Natl Bur Stand (US).

[17] Wapstra AH, Audi G (1985). Nucl Phys.

[18] White JR, Cameron AE (1948). Phys Rev.

[19] Baxter GP, Parsons LW (1921). J Amer Chem Soc.

[20] Baxter GP, Hilton FA (1923). J Amer Chem Soc.

[21] Baxter GP, Ishimaru S (1929). J Amer Chem Soc.

[22] Baxter GP, Dorcas MJ (1924). J Amer Chem Soc.

[23] Barnes IL, Garner EL, Gramlich JW, Machlan LA, Moody JR, Moore LJ, Murphy TJ, Shields WR (1973). Geochim Cosmochim Acta Suppl 4.

[24] (1988). Atomic weights of the elements 1987. Pure Appl Chem.

[25] Peiser HS (1985). Anal Chem.

[26] Kuehner EC, Alvarez RA, Paulsen PJ, Murphy TJ (1972). Anal Chem.

[b27-jresv94n6p347_a1b] 27Shideler, R. W., and Gramlich, J. W., in preparation.

